# Evaluation of a programmable analyser – the Vitatron PA800

**DOI:** 10.1155/S1463924681000369

**Published:** 1981

**Authors:** Janet M. Smith, I. Fry, A. W. Walker

**Affiliations:** Department of Clinical Biochemistry St. Luke's Hospital Surrey Guildford UK


[3] Eck, G. F., 1970, Performance of a process stream analyser in a Sun Oil complex, *Instrument Society of America*, **25**, 740
[4] Topham, W. H., 1970, New approach to gas chromatographic systems for process control, *Instrument Society of America*, **25**, part III, 749
[5] Maselli, S., 1972, Control both ends for profit, *Instrument Society of America*, **27**, part IV, 831
[6] Clinton, R. M. and Puzniak, T. J., 1975, *Instrument Society of America*, **22**, 47
[7] Read, B. J. and Williamson, J. A., 1975, On-line analysis, *Control and Instrumentation*, 7, 6, 18-21
[8] US Patents 3 622 489 and 3 622 488, Dynascience Corp, Los Angeles, California, USA

### Evaluation of a programmable analyser – the Vitatron PA800

**Janet M. Smith, I. Fry and A. W. Walker**
*Department of Clinical Biochemistry, St. Luke's Hospital, Guildford, Surrey, UK.*

### Introduction

Versatile analysers capable of measuring serum constituents by different principles are gaining increasing popularity in the clinical laboratory. To compete with or complement large multichannel instruments, they must be efficient in terms of running costs, labour costs and speed as well as producing accurate and precise results. The Vitatron programmable analyser (PA800) was evaluated using methods for frequently requested analyses, aspartate transaminase (AST), total bilirubin, cholesterol and glucose as well as for the measurement of phenytoin by the EMIT* technique. The methods were chosen to permit the instrument to display its versatility regarding measuring principles. The evaluation was carried out for the Department of Health and Social Security and the protocol was based on the recommendations of Broughton *et al* [1].

### The Vitatron PA800

The instrument is the successor to the Vitatron Automatic Kinetic Enzyme System (AKES), a discrete analyser, evaluation reports of which have been published elsewhere [2,3]. In addition to performing kinetic analysis of both enzymes and substrates, the new instrument can also perform endpoint analysis. In addition, the computer is capable of curve fitting which increases the range of analyses which can be performed by the instrument. Externally, the PA800 looks very similar to the AKES but there are several differences in its mode of operation. The principle of the transport mechanism, the movement of coded sample holders in a chain through the system synchronised to the rotation of a ring of eighteen thermostatted glass cuvettes through the dispensing points and the lightpath, is unaltered. However, whereas the coded sample holders for the AKES were supplied in chains of ten those for the PA800 are supplied individually. Unnecessary sample holders are not put through the system which improves the instrument efficiency.

The PA800 uses reagents economically, they are sampled from a compartmentalised reagent tray through stainless steel probes in the sampling head by a water–filled syringe system. The disposable reagent trays have two compartments, a smaller inner compartment for 'starter' reagent and a large one for the diluent reagent. For use, the tray is sited adjacent to the cuvette ring. Three separate probes, one each for sample, diluent and starter, are incorporated into the sampling head. Cleaning solution can also be dispensed from each probe into the cuvettes and, allows three cuvette washes between analyses and as a vibrating mixer for the cuvette contents (when reagent or sample are dispensed into a cuvette). Volumes picked up and dispensed through the probes are selected manually by means of micrometer screws on Hamilton syringes. A stainless steel suction tube, positioned in the sampling head, removes the cell contents, under vaccuum, to a waste unit sited behind the sampling head. The photometer has a quartz-iodine lamp and the wavelength is selected by means of interference filters. Two integrators are built into the photometer. During the measurement of absorbance changes, ie whilst one integrator is performing an integration, the other is transferring the results of the previous integration to the calculator.

The system of audio and visual warnings used in the AKES has been incorporated into the PA800, the audible warning drawing attention to the instrument and the warning lamps directing the operator to the source of the problem. In addition, a temperature warning lamp is illuminated if the cuvette ring is not at the programmed temperature.

The analytical process is controlled by the Canon Canola SX - III calculator, which is an integral part of the system. Instructions are fed into the calculator via magnetic cards. Two general program cards are fed in daily. One or more method cards, containing instructions specific for the assay to be performed, including temperature of assay, incubation times, number of measuring points and mode of calculation, are fed in before performing an assay. Information and results are printed on a tally roll. When a method program card has been loaded, the syringe settings and interference filter required are printed by the calculator and adjustments are made manually. Alteration of the diaphragm position on the photometer is also performed manually, but blanking is carried out automatically by the instrument.

The mathematical analysis of the absorbance data for kinetic measurements on the PA800 is more sophisticated than that on the AKES. The photometer integrates the absorbance change over consecutive intervals of 0.287 seconds, the number of readings being programmed for the method in question. These measuring points are divided into four blocks and the sum of integrations within each block is calculated. After the monitoring period, the slope of the line is calculated at two points during the time course of the

* *EMIT is a registered trademark of the Syva Corporation.*

reaction, using the absorbance changes in all four blocks for calculation. The overall slope is computed from these figures and the result calculated from this. In the case of enzyme activity analysis, the two slope measurements are compared to give a percentage deviation from linearity, which is printed alongside the result.

For the endpoint measurements, the mean final absorbance value is calculated from the readings taken at 0.287 second intervals. This is done initially for each of the eighteen cuvettes to give individual cuvette blank readings, which are stored by the computer and subtracted appropriately from all the subsequent absorbance readings, along with the reagent blank value, which is derived from three aliquots of reagent. The mean absorbance values for three aliquots of the same standard serum are measured and used to compute the factor necessary for result calculation. There is an alternative facility where this factor is pre-programmed into the computer, obviating the need to put standards through with each batch. Where sample blanks are required, the sera are run through the instrument with suitable diluent prior to the run with reagents, the blank readings being stored by the computer for subtraction from the final result.

For the calculation of results for EMIT analyses, a response is calculated for each sample or standard, relative to the absorbance change measured for the zero standard. Several standards are put through the system and the calculator processes the responses to give a log-logit equation. The sample results are calculated from this equation. For thyroxine measurement by the EMIT technique, a modified Gauss-Newton process is used for curve-fitting. For turbidimetric analyses, at least three standards of different concentration are processed by the instrument and the constants for the curve equation are derived by the computer. We have used a kit for IgG measurement, manufactured by the Boehringer Corporation, which is supplied with only three standards, and have obtained satisfactory results using the PA800. However, where possible, more than three standards should be processed to define the standard curve more clearly.

An optional extra component of the system is a Vitatron 2001 series recorder on which the photometer output is expressed graphically. This provides valuable additional information, particularly for enzyme activity measurements where there are problems with the chemistry of the reaction, such as unacceptable deviation from linearity or substrate exhaustion.

### Materials and methods

The evaluation was carried out over a 4 month period. Four methods were examined in detail by the following procedures.

(a) *Precision* A 20-day precision study, involving the analysis of 20 aliquots of each of three quality control sera with different concentrations of the constituent in question on each of the 20 days, was carried out.

(b) *Accuracy* The main assessment of accuracy was by comparison of results obtained using the PA800 on at least 200 samples with those obtained using methods in routine use in this laboratory. Analysis of commercially prepared quality control sera with assigned values for the constituents studied was also performed.

(c) *Linearity* Two samples, one of high concentration and one of low concentration were combined to give samples with a range of concentration which were analysed and the results compared with the expected values.

(d) *'Carryover'* Studies to assess the contribution of 'carryover' to results were carried out by repeated analysis of alternate high and low concentration samples in triplicate, as described by Young and Gochman [4].

The methodologies studied with this protocol and those used for comparative purposes were as follows:

*Aspartate transaminase* This was measured according to the recommendations of the Committee on Enzymes of the Scandinavian Society for Clinical Biochemistry and Clinical Physiology [5] and the assay was compared with the same method performed on the Vitatron AKES. On both instruments, the reaction took place at 37°C and was monitored at 340 nm. Reagents were manufactured by J. T. Baker Chemicals, Deventer, Holland and supplied by Diamed Diagnostics, Merseyside, U.K.

*Total bilirubin* Plasma bilirubin was measured by the method of Jendrassik [6], by endpoint analysis with serum blank correction, using a temperature of 37°C and a wavelength of 546 nm. Reagents were supplied by the Boehringer Corporation (London) Ltd. For comparative purposes, bilirubin was measured according to the method of Michaelsson [7] and the recommendations of Billing, Haslam and Wald [8].

*Cholesterol* Plasma cholesterol was measured by kinetic substrate analysis, without a starter reagent, using a cholesterol oxidase method. The hydrogen peroxide produced oxidises methanol to formaldehyde which reacts with ammonium ions and acetylacetone to produce a dihydrolutidine derivative which absorbs at 410 nm [9]. The assay was performed at 37°C. Reagents were supplied by the Boehringer Corporation. The method was compared to another cholesterol oxidase method, using Trinder's chromogen system [10], performed on the Vickers M300 multichannel analyser [11].

*Glucose* Plasma glucose was measured by kinetic substrate analysis with a starter reagent, using glucose oxidase cleavage with the Trinder chromogen system [10]. The colour was measured at 546 nm and the assay performed at 25°C. Reagents were supplied by the Boehringer Corporation. For comparative purposes, glucose was measured on fluoridised plasma samples by the glucose analyser, model 23AM of the Yellow Springs Instrument Co. Ltd., which measures electrochemically the hydrogen peroxide produced from glucose by the action of immobilised glucose oxidase. This instrument operates at 37°C.

Less extensive studies were performed on two further methods:

*Cholesterol* A cholesterol oxidase method, using endpoint analysis, was examined. The hydrogen peroxide produced converts iodide to iodine, which is measured photometrically

**Table 1. Alteration of temperature setting**

| Initial Temperature setting °C. | Final Temperature setting °C. | Time taken to achieve new setting (mins.) |
|---|---|---|
| 22.7 (Room Temperature) | 30.0 | 3.9 |
| 25.0 | 30.0 | 2.4 |
| 25.0 | 37.0 | 5.8 |
| 30.0 | 25.0 | 3.6 |
| 30.0 | 37.0 | 3.7 |
| 37.0 | 25.0 | 6.4 |
| 37.0 | 30.0 | 3.2 |

**Table 2. Assay costs and rate of analysis**

| Assay | Mode of analysis | Rate of Analysis (per hour) | Cost per Test (p.) |
|---|---|---|---|
| Aspartate transaminase | Kinetic | 144 | 1.7 |
| Total bilirubin | Endpoint with serum blank | 65 | 2.4 |
| Cholesterol | Kinetic | 120 | 15.5 |
| Glucose | Kinetic with 'Starter' | 180 | 0.1 |
| Cholesterol | Endpoint | 90 | 11.0 |
| Phenytoin | Curve Analysis | 144 | 72.6 |

at 356 nm. The reagents were manufactured by E. Merck, Darmstadt, Federal Republic of Germany and supplied by BDH Chemicals, Poole, U.K. Comparative studies were carried out using the Vickers M300 method described above [11].

*Phenytoin* Phenytoin was measured using the EMIT technique [12] with reagents supplied by Syva Diagnostics (UK) Ltd, Maidenhead. The reaction was monitored at 340 nm and performed at 30°C. A 4 µl sample of neat serum was added to a diluent solution comprising the enzyme-labelled drug in a Tris-HCl buffer. The reaction was initiated, prior to monitoring, with a concentrated solution containing antibody to the drug, enzyme, substrate and coenzyme (NAD).

Quality control sera used for the study were supplied by the Boehringer Corporation, Wellcome Reagents Ltd, London, and BDH Chemicals.

### Results and discussion

The PA800 is simple to use and economical on labour. The instrument can be run efficiently by one operator who can be usefully employed preparing reagents and samples for the subsequent assay or processing results from the previous assay while the instrument is in operation. The changeover time from one assay to another is minimal, the rate-limiting factor being the time taken for the cuvette ring to achieve the programmed temperature (Table 1). The speed of assay depends on the particular methodology with a maximum of 180 and an average of 120 samples being processed per hour (Table 2). The cost per test, assuming a batch size of 50 samples, is shown in Table 2. These costs are reasonable and although the phenytoin cost appears high, it is only 70% of using the technique manually.

The performance of assays on the instrument depends on the accuracy and reproducibility of instrumental components. The temperature control and syringe precision were studied as well as several features of the photometer.

### Temperature control

External temperature measurements were made with a Digitherm Mark II digital thermometer, manufactured by Kane May (Barrow Field, Welwyn Garden City, Herts, U.K.), which was inserted into the heating block surrounding the cuvettes for readings to be taken. The time taken to achieve the set temperature after re-programming the instrument was measured in this way (Table 1), as was the temperature stability at the three set temperatures (Table 3). It was not possible to assess the accuracy of the thermostat by this method, since the accuracy of the thermometer was unknown. A temperature sensor is fitted into the instrument and the calculator can be instructed to print the temperature at any time. Vitatron claim that this sensor is extremely accurate and the temperature printed is the true temperature of the cuvette ring. Using this facility no temperature drift was observed during assays.

### Syringe precision

No satisfactory way could be found to test the precision of the syringes individually because the overall precision of the sampling and dispensing system depends on the mixing and cleaning features which do not function unless the instrument is in operation. Consequently, the syringe combinations of 'Diluent-syringe with Starter syringe' and 'Diluent syringe with Sample syringe' were tested using solutions of potassium dichromate in 0.01N $H_2SO_4$ as the 'starter' or 'sample' respectively and 0.01N $H_2SO_4$ as diluent. Several combinations of volumes were examined, the concentration of the potassium dichromate solutions being adjusted to give a final cuvette concentration of 0.01g/100ml. The absorbances of the resultant solutions were in the order of 1.00 and were measured at 366 nm. Using an arbitrary figure as the standard value and an endpoint programme, it was possible to obtain values from which the syringe precisions could be calculated. The results are shown in Table 4 and are satisfactory. Studies outside the evaluation, using smaller volumes down to a 4 µl sample size, which is used for several methods on the instrument, have shown the precision (CV%) to be in the order of 1.5.

### Photometer studies

*Filter characteristics*: Five filters supplied with the instrument were examined using an SP1800 recording spectrophotometer. Each filter was scanned between 200 and 600 nm and the wavelength at which the maximum transmission occurred was noted. The half-band width was calculated for this peak. It was ascertained that only one transmission peak was present for each filter. Results for the filter studies are shown in Table 5. The spectral quality of the filters was satisfactory although the half-band width of the 340 and 360 nm filters were marginally outside the specifications of the manufacturer (8 - 12 nm).

*Precision*: The precision of the photometer was studied at 366 nm by taking multiple absorbance readings of potassium

**Table 3. Temperature stability**

| Temperature setting | 25°C | 30°C | 37°C |
|---|---|---|---|
| No of digitherm readings (at 1 minute intervals) | 11 | 11 | 11 |
| Mean digitherm reading | 26.40 | 31.08 | 38.31 |
| SD | 0.05 | 0.06 | 0.03 |
| CV% | 0.18 | 0.19 | 0.08 |

**Table 4. Syringe precision**

| Diluent syringe volume µl | Starter syringe volume µl | Sample syringe volume | N | Mean Reading | SD | CV % |
|---|---|---|---|---|---|---|
| 700 | 10 | – | 40 | 151.37 | 0.881 | 0.58 |
| 600 | 50 | – | 40 | 149.09 | 1.097 | 0.74 |
| 600 | 100 | – | 40 | 149.67 | 0.84 | 0.56 |
| 700 | – | 10 | 40 | 100.38 | 1.305 | 1.30 |
| 600 | – | 100 | 31 | 102.16 | 0.479 | 0.47 |

**Table 5. Filter studies**

| Assigned wavelength of filter nm. | Wavelength at maximum transmission nm. | Half bandwidth nm. |
|---|---|---|
| 340 | 340 | 12.8 |
| 366 | 366 | 12.5 |
| 405 | 406 | 10.5 |
| 490 | 491 | 9.0 |
| 546 | 544 | 10.0 |

**Table 6. Photometer precision**

| $K_2Cr_2O_7$ concentration mg/1 | N | Mean absorbance reading | SD | CV % |
|---|---|---|---|---|
| 250 | 10 | 1.984 | 0.006 | 0.29 |
| 190 | 10 | 1.573 | 0.005 | 0.32 |
| 125 | 20 | 1.022 | 0.002 | 0.25 |
| 94 | 20 | 0.760 | 0.003 | 0.36 |
| 62.5 | 20 | 0.489 | 0.002 | 0.51 |
| 31.3 | 20 | 0.251 | 0.003 | 1.08 |
| 15.7 | 20 | 0.125 | 0.001 | 0.91 |
| 10.4 | 20 | 0.081 | 0.002 | 2.12 |

dichromate solutions with a range of concentration. The precision of the absorbance values throughout the range studied was satisfactory (Table 6).

*Linearity*: The linearity of the photometer response was studied with a potassium dichromate solution at 366 nm, a nickel sulphate solution at 405 nm and a cobalt nitrate solution at 546 nm. The response was found to be linear at the three wavelengths, all measured absorbances falling close to their expected values. The authors were particularly impressed with the linearity at very low absorbance values (Figure 1).

### Methodological studies

### Precision

The results of the 20-day precision study are shown in Tables 7 and 8. The final values obtained for the coefficients of variation for all methods were acceptable.

It will be noted that the between-batch coefficients of variation for cholesterol, measured kinetically, and glucose are closer in magnitude to the within-batch figures than might be expected. The authors were disappointed with the within-batch precision figures achieved during the 20-day study and obtain much lower figures during routine operation of the instrument. The precision figures we obtain between-batch are similar to those the authors found during the 20-day period. Our data suggests that the unexpectedly high coefficient of variation figures found within-batch reflect relatively poor precision on one or two days during the 20-day study.As the mean value for the concentration of the constituents on those days was not siginificantly different from the overall mean, such imprecision does not affect the between-batch figures.

The precision figures for the endpoint cholesterol method are shown in Table 9. The CV% values found in both within-batch and between-batch studies are better than those found with the kinetic cholesterol method but it should be noted that the endpoint method was not subjected to such a rigorous precision study and was studied when the operator had gained considerable experience with the instrument. The endpoint method was preferred because the reagent preparation was easier and because it was cheaper (Table 1). The precision figures for the other kinetic method studied, glucose, are better than those for cholesterol probably because the reaction is much faster and the absorbance changes being measured are greater, the reaction being initiated with a starter reagent just before the monitoring position. A kinetic uric acid method is routinely used on the instrument in which uricase is added as the starter reagent, this performs completely satisfactorily. It is recommended that where substrate measurement is being contemplated on

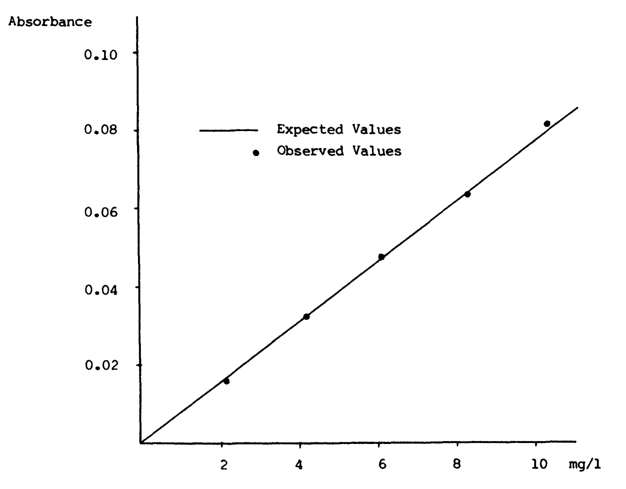

*Figure 1. Photometer Linearity 366nm*

**Table 7. Within-batch precision**

| Serum constituent | Level | Grand mean | Mean of SD | Mean of CV% |
|---|---|---|---|---|
| Aspartate transaminase U/1 | Low | 22.52 | 0.87 | 3.80 |
| | Medium | 49.10 | 1.15 | 2.33 |
| | High | 167.06 | 2.10 | 1.24 |
| Bilirubin µmol/1 | Low | 3.47 | 0.32 | 9.70 |
| | Medium | 19.42 | 0.77 | 3.87 |
| | High | 74.46 | 2.14 | 2.89 |
| Cholesterol mmol/1 | Low | 2.69 | 0.20 | 7.19 |
| | Medium | 5.96 | 0.26 | 4.40 |
| | High | 8.82 | 0.38 | 4.26 |
| Glucose mmol/1 | Low | 2.53 | 0.12 | 5.08 |
| | Medium | ·6.05 | 0.14 | 2.27 |
| | High | 12.00 | 0.25 | 2.06 |

**Table 8. Between-batch precision**

| Serum constituent | Level | Grand mean | SD of day means | CV % |
|---|---|---|---|---|
| Aspartate transaminase U/1 | Low | 22.52 | 1.85 | 8.22 |
| | Medium | 49.10 | 2.33 | 4.74 |
| | High | 167.06 | 4.21 | 2.52 |
| Bilirubin µmol/1 | Low | 3.47 | 0.53 | 15.32 |
| | Medium | 19.42 | 1.43 | 7.35 |
| | High | 74.46 | 4.22 | 5.66 |
| Cholesterol mmol/1 | Low | 2.69 | 0.17 | 6.17 |
| | Medium | 5.96 | 0.28 | 4.68 |
| | High | 8.82 | 0.40 | 4.51 |
| Glucose mmol/1 | Low | 2.53 | 0.12 | 4.63 |
| | Medium | 6.05 | 0.14 | 2.33 |
| | High | 12.00 | 0.34 | 2.84 |

**Table 9. Cholesterol (endpoint) precision study**

| | Sample | N | Mean mmol/1 | SD mmol/1 | CV % |
|---|---|---|---|---|---|
| Within-batch | Serum A | 20 | 2.765 | 0.049 | 1.77 |
| | Serum B | 20 | 5.490 | 0.079 | 1.44 |
| | Serum C | 20 | 6.835 | 0.093 | 1.37 |
| Between-batch | Precilip | 21 | 3.429 | 0.085 | 2.47 |
| | Seronorm lipid | 20 | 6.180 | 0.144 | 2.32 |
| | Precilip EL | 20 | 7.480 | 0.104 | 1.39 |

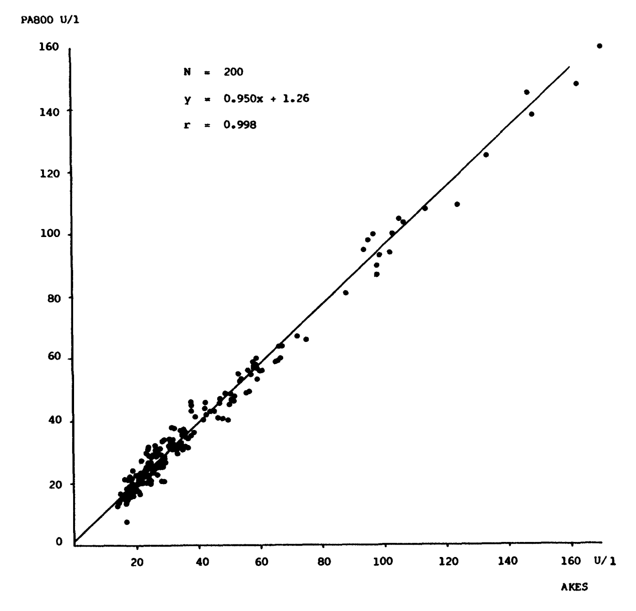

*Figure 2. Accuracy: Aspartate Transaminase*

the PA800, an endpoint method or a kinetic method which is fast and utilises a starter reagent, should be chosen. Precision figures for the phenytoin assay are shown in Table 10. These were calculated by statistical analysis of the results obtained on duplicate samples assayed in the same batch for within-batch figures and in different batches for between-batch figures. The CV% values are far better than those generally accepted for immunoassay techniques.

### Accuracy

Results of the comparative studies with alternative methods are shown in Figures 2 - 6 and results obtained on quality control sera are shown in Table 11. The authors were pleased with the performance of all assays with respect to accuracy, the correlation coefficients being above 0.99 for all methods except the kinetic measurement of cholesterol. Results obtained from the glucose assay were lower than those obtained by the Yellow Springs Glucose analyser. Results obtained on a continuous flow system using the Trinder method are lower than those measured on the Yellow Springs instrument and presumably this is a feature of the different principles of measurement. It will be noted that results from the endpoint cholesterol method are lower than those obtained on the Vickers M300 (Figure 6). This method is one for which a pre-programmed factor for result calculation is employed and we found all results to be too low as judged by the comparative method and by values obtained on quality control sera with assigned values. Subsequently the factor was changed from 8.15 to 9.0 and this led to much better agreement with alternative methods. The results in Table 11 for this method were obtained after the factor had been altered. The other endpoint method studied, bilirubin, was also found to be inaccurate using the programmed factor and this was changed from 146 to 175 to produce acceptable results. The factor of 175 was used throughout the evaluation period. It is recommended that where possible, serum standards are analysed with endpoint assays, allowing the factor to be calculated for each run of samples.

### Linearity

The results of the linearity studies are shown in Table 12. All methods were linear throughout the ranges studied which in all cases extended to pathological levels. Comparative studies using patient samples have shown that the bilirubin and the kinetic cholesterol methods are not linear above 200μmol/l and 10mmol/l respectively.

### Carryover

The figures obtained for mean percentage interaction (I%) for the methods studied were:- aspartate transaminase 0.0, bilirubin 0.11, cholesterol 0.94 and glucose 0.10. These are all well below the maximum recommended figure of 2% [4], as would be expected with the discrete sampling mechanism and adequate washing of the sample probe.

Apart from the problems with pre-programmed factors mentioned earlier, all the kinetic and endpoint methods studied functioned well on the PA800 when the recommendations of the instrument manufacturer were followed. However problems were found with the EMIT Phenytoin assay and the method had to be modified before acceptable results were produced. The reagent preparation was altered such that the NAD was in the starter reagent, not the diluent solution, in which it was unstable.

**Table 10. Phenytoin precision study**

| | No. of pairs | Range of Values μg/ml | Mean Value μg/100ml | SD μg/100ml | CV % |
|---|---|---|---|---|---|
| Within-batch | 20 | 0.9–33.5 | 11.86 | 0.523 | 4.4 |
| Between batch | 28 | 0.4–36.8 | 10.84 | 0.367 | 3.4 |

**Table 11. Accuracy – control sera studies**

| Assay | Material used | No. of analyses | Mean result | Assigned value |
|---|---|---|---|---|
| Aspartate | Wellcomtrol $BC0_2$ | 21 | 162 | 167 |
| Transaminase | Precinorm U | 7 | 58 | 60 |
| U/l | Precipath E | 4 | 138 | 142 |
| Bilirubin | Wellcomtrol $BC0_2$ | 16 | 89.5 | 88 |
| μmol/l | Pricinorm U | 18 | 20 | 23 |
| Cholesterol | Precilip | 9 | 3.8 | 3.2 |
| (Kinetic) | Precilip E.L. | 6 | 8.2 | 7.5 |
| mmol/l | | | | |
| Glucose | Wellcomtrol $BC0_2$ | 18 | 10.9 | 11.8 |
| mmol/l | Wellcomtrol $BC0_3$ | 15 | 1.9 | 3.1 |
| Cholesterol | Precilip | 21 | 3.4 | 3.2 |
| (endpoint) | Seronorm Lipid | 20 | 6.2 | 6.5 |
| mmol/l | Pricilip E.L. | 20 | 7.5 | 7.5 |
| Phenytoin | Wellcomtrol $BC0_3$ | 15 | 12.9 | 12.6 |
| μg/ml | | | | |

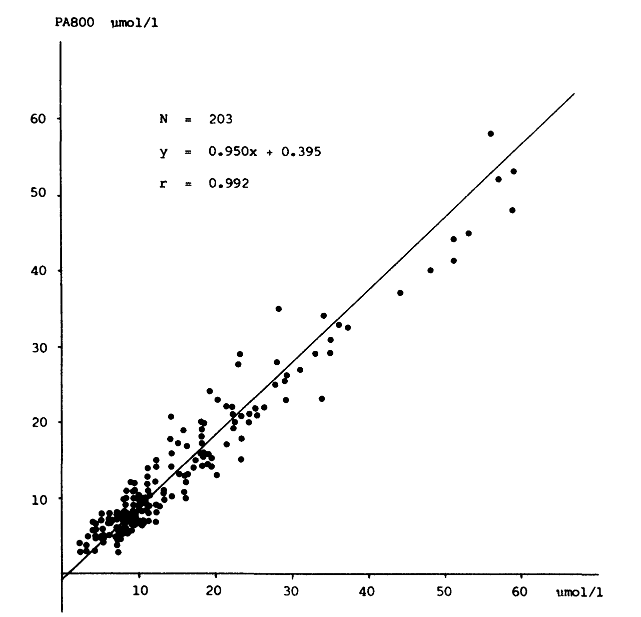

*Figure 3. Accuracy: Bilirubin*

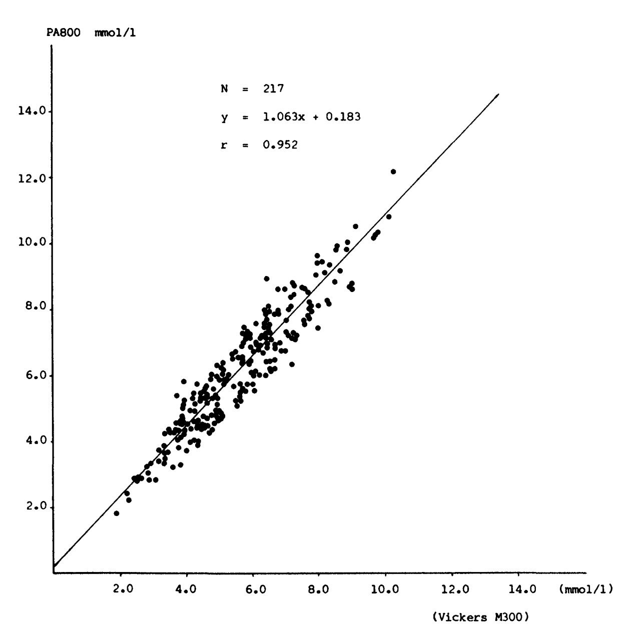

*Figure 4. Accuracy: Cholesterol (Kinetic)*

The criticisms of design made in the report on the AKES [2] have largely been overcome on the PA800. Although there is still no choice in the position at which starter reagent can be added, the improved method of calculation of enzyme activity ensures that, should a reaction be initiated by serum as for creatine phosphokinase measurement, the incubation times for all cuvettes are identical, which was not the case on the AKES. Similarly, where a starter reagent is used, the incubation period with starter, before the reaction is monitored, is identical for all cuvettes. The programmable calculator gives flexibility ie incubation times and programme changes can be effected simply by the operator. A fast transport mechanism has been incorporated into the instrument so that, if required, a chain of sample holders can be removed rapidly from the system. A modification has been made to the cuvette ring. The cuvettes in the PA800 are detachable from their mounting so that should one need to be replaced, it can be changed easily by the operator. Formerly, the cuvettes were fixed in their mounting and damage to one cuvette necessitated returning the complete ring to the manufacturer for repair.

The authors have some minor criticisms of the instrument. Firstly, the silicone tubing which carries the cuvette contents to the waste unit is directed around the back of the sampling head and great care must be exercised when lowering the sampling head into position to prevent the tubing being trapped. Secondly, when a leak occurs in a part of the system not immediately visible, there is no alarm and the presence of the leak is not made obvious. This is a particular problem if tubing became detached inside the master value unit or inside the base of the waste unit.

Several faults occurred on the instrument used during the first six months it was in the laboratory. All were rectified promptly by the service engineer. The most worrying fault concerned the temperature control system and was eventually traced to a faulty transistor. There was no warning of the existence of this fault as the temperature warning light was not illuminated, even though the cuvette ring had not achieved the programme temperature. Similary, when the 'temperature' key on the calculator was depressed the programmed, not the actual, temperature was printed. The problem has not recurred since the transistor was replaced.

The instrument was supplied with a belt-driven sampling-head mechanism, which failed on three occasions due to breakage of the drive-belt and the instrument has since been fitted with a spindle-driven mechanism, analagous to that on the 'AKES. All new models are being supplied with this mechanism and those already installed have been modified. Three other minor faults were swiftly rectified. These were caused by a defective drive belt on one of the mixing probes such that it could not vibrate, a loose electrical connection in the sampling head and a faulty flange in a tubing connection. The instrument was subjected to an electrical safety examination and was found to comply with the Electrical Safety Code for Hospital Laboratory Equipment.

No problems were experienced with the calculator which greatly improves the efficiency of the analytical process.

In certain laboratory situations a larger computer would be an advantage. If memory were to be increased, additional versatility and improvements would be possible. For example, absorbance readings could be stored until the end of a run for scrutiny before result calculation. Long term storage of data would allow results of several analyses on one patient to be reported together, reducing the potential for

**Table 12. Linearity studies**

| % A in specimen | Aspartate transaminase U/l | | Bilirubin µmol/l | | Cholesterol mmol/l | | Glucose mmol/l | |
|---|---|---|---|---|---|---|---|---|
| | Expected concentration | Observed concentration | Expected | Observed | Expected | Observed | Expected | Observed |
| 0 | 26.5 | | 1.80 | | 1.55 | | 1.96 | |
| 20 | 53.7 | 53.3 | 34.09 | 31.85 | 3.03 | 3.05 | 7.12 | 6.97 |
| 40 | 80.9 | 80.0 | 66.38 | 63.03 | 4.50 | 4.48 | 12.28 | 12.24 |
| 60 | 108.1 | 107.3 | 98.67 | 93.63 | 5.98 | 6.15 | 17.43 | 17.94 |
| 80 | 135.3 | 134.0 | 130.96 | 131.38 | 7.45 | 7.48 | 22.59 | 22.84 |
| 100 | 162.5 | | 163.25 | | 8.93 | | 27.75 | |

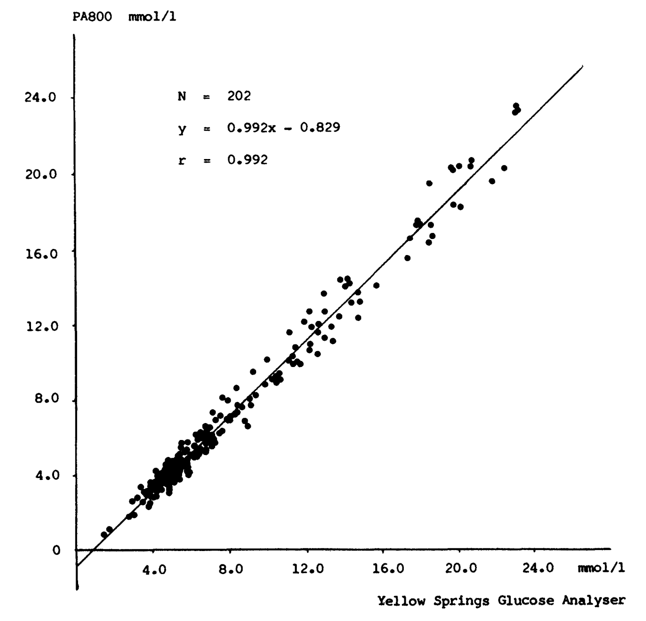

*Figure 5. Accuracy: Glucose*

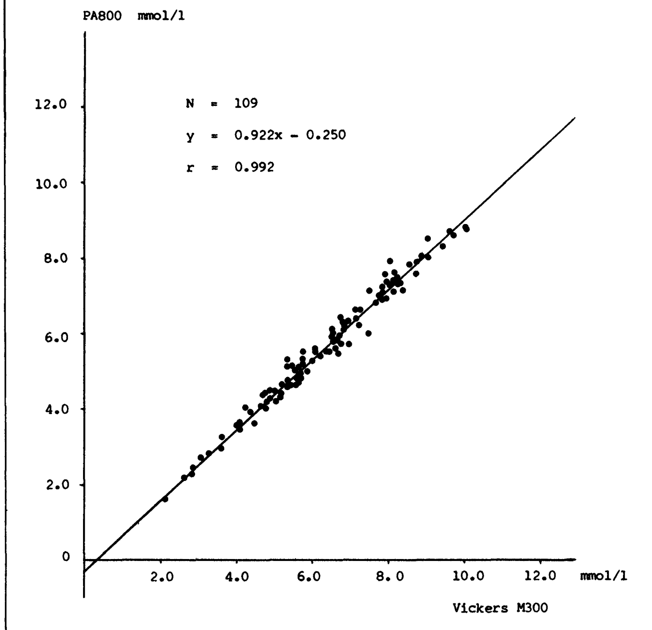

*Figure 6. Accuracy: Cholesterol (Endpoint)*

reporting errors. An increased computer facility would be particularly useful for laboratories which do not have a sophisticated data-processing system. In its present form, the output of the calculator can be fed directly into data processing systems in current use in clinical biochemistry.

### Conclusions

The Vitatron PA800 is a reliable instrument which produces accurate results on small volumes of serum with minimum wastage of reagents. It is equally suitable for the analysis of large batches of samples for the more commonly requested clinical analyses and for processing small numbers of samples, whether for more specialised analyses or in a laboratory with a small workload. This versatility should enable the instrument to fulfil a useful role in most clinical laboratories.

ACKNOWLEDGEMENTS
The authors would like to thank Mr. Ian Martin for performing the statistical analysis of the precision data, the DHSS and MSE Scientific Instruments, who jointly financed the Evaluation and Mrs. Linda Holmes for secretarial assistance.

REFERENCES

[1] Broughton, P. M. G., Gowenlock, A. H., McCormack, J. J., and Neill, D. W. (1974). Revised scheme for the evaluation of automatic instruments in Clinical Chemistry. *Ann. Clin. Biochem.* **11**, 207-218.

[2] Patel, Shanta and O'Gorman P. (1975). Assessment of a new enzyme reaction rate analyser, the Vitatron AKES. *Clin. Chim. Acta* **60**, 249-258.

[3] McQueen, M. J., King, J. (1957). Evaluation of the Vitatron Automatic Kinetic Enzyme System. *Clin. Chim. Acta* **64**, 155-164.

[4] Young, D. S., and Gochman, N. (1972). Methods for assuring quality data from continous flow analysers. *Stand. Methods in Clin. Chem.* 7, 293-304.

[5] The Committee on Enzymes of the Scandinavian Society for Clinical Chemistry and Clinical Physiology (1974). Recommended methods for the determination of four enzymes in blood. *Scand. J. Clin. Lab. Invest.* **33**, 291-305.

[6] Jendrassik, L. and Grőf, P. (1938). *Vereinfachte photometrische Methoden zur Bestimmung des Blutbilirubins. Biochem Z.* **297**, 81-89.

[7] Michaelsson M., Nosslin, B. and Sjölin, S. (1965). Plasma bilirubin determination in the newborn infant. *Pediatrics* **35**, 925-931.

[8] Billing, Barbara, Haslam, Ruth and Wald, N. (1971). Bilirubin standards and the determination of bilirubin by manual and Technicon AutoAnalyser methods. *Ann. Clin. Biochem.* **8**, 21-30.

[9] Röschlau, P., Bernt, E. and Gruber, W. (1974). Enzymatic determination of total cholesterol in serum. *Z. Klin. Chem. Klin. Biochem.* **12**, 403-407.

[10] Trinder, P., (1969). Determination of glucose in blood using glucose oxidase with an alternative oxygen acceptor. *Ann. Clin. Biochem.* **6**, 24-27.

[11] Nobbs, B. T., Smith, Janet M. and Walker, A. W. (1977). Enzymic determination of plasma cholesterol on discrete automatic analysers. *Clin. Chim. Acta* **79**, 391-397.

[12] Rubenstein, K. E., Schneider, R. S. and Ullman E. F. (1972). Homogeneous Enzyme Immunoassay. A new immunochemical technique. *Biochem. Biophys. Res. Commun.* **47**, 846-851.

### An evaluation of the Chemispek multichannel analyser

**R.W. Logan*, A.K. Tweedie, Evelyn M. Aitchison and Elizabeth C. Jamieson**
*Department of Biochemistry, Yorkhill Children's and Maternity Hospitals, Glasgow, UK.*

### Description

The Chemispek is a multichannel analyser of the continuous flow type with a microprocessor-based system for collection and presentation of results. Modular in design, the equipment is similar in many respects to the earlier Chromaspek amino acid analyser produced by the same company (Rank Hilger, Margate).

At present, up to twelve simultaneous channels may be operated, chosen from a range of seventeen chemistries.

An eight channel configuration was chosen as being most suitable for the requirements of this department providing the following analyses: sodium, potassium, chloride, carbon dioxide, urea, creatinine, calcium and phosphate.

The instrument is supplied complete with a mounting stand although the individual modules may be bench mounted. The dimensions are length, 2.5 m; depth 0.8 m and height, 1.5 m. An additional stand carrying a chart recorder and microprocessor measures 0.8 m in length, 0.8 m in depth and 1 m in height. There is also a free standing matrix printer of similar dimensions to the smaller stand.

**Correspondence should be addressed to Dr. R.W. Logan, Consultant Medical Biochemist at the above address.*

The instrument is designed to be operated at sampling speeds of up to 120/h depending upon the chemistries involved. As no system of positive sample identification is provided, it is necessary to record the exact sequence of analysis of the specimens in the trays. The sampler module provides for switch-selectable sampling rates covering a wide range of settings. The sample to wash ratio is variable and there is a facility to introduce an air bubble between samples.

Reagents are distributed to each twin channel chemistry module by means of four-speed peristaltic pumps. Wash valves provide the means by which all or individual channels may be switched to wash water. The frequency of air injection to the flowing reagent stream may be altered by thumbscrews mounted on the introduction blocks. The fast speed setting of the pumps allows for rapid filling of the system with reagent or fast wash out with water, while the slowest pump speed setting allows the machine to be kept in a state of readiness over long periods while using a minimum of reagent.

Heating baths, dialysers, coils and connecting tubing are mounted on removable plastic trays. Effluent is channelled away through a series of interconnected drains which may be

